# What Fechner could not do: Separating perceptual encoding and decoding with difference scaling

**DOI:** 10.1167/jov.24.5.5

**Published:** 2024-05-09

**Authors:** Joris Vincent, Marianne Maertens, Guillermo Aguilar

**Affiliations:** 1 Computational Psychology, Technische Universität, Berlin, Germany

**Keywords:** brightness, lightness, scaling, MLCM, matching, encoding functions, transfer functions, White’s illusion

## Abstract

A key question in perception research is how stimulus variations translate into perceptual magnitudes, that is, the perceptual *encoding* process. As experimenters, we cannot probe perceptual magnitudes directly, but infer the encoding process from responses obtained in a psychophysical experiment. The most prominent experimental technique to measure perceptual appearance is matching, where observers adjust a probe stimulus to match a target in its appearance along the dimension of interest. The resulting data quantify the perceived magnitude of the target in physical units of the probe, and are thus an indirect expression of the underlying encoding process. In this paper, we show analytically and in simulation that data from matching tasks do not sufficiently constrain perceptual encoding functions, because there exist an infinite number of pairs of encoding functions that generate the same matching data. We use simulation to demonstrate that maximum likelihood conjoint measurement (Ho, Landy, & Maloney, 2008; Knoblauch & Maloney, 2012) does an excellent job of recovering the shape of ground truth encoding functions from data that were generated with these very functions. Finally, we measure perceptual scales and matching data for White’s effect (White, 1979) and show that the matching data can be predicted from the estimated encoding functions, down to individual differences.

## Introduction

As psychophysicists we study human visual perception using a black box approach (Georgeson, 1979). We systematically vary the input to the visual system along some stimulus dimension of interest (*S*), and measure the corresponding output, that is, the behavioral response (*R*). If the chosen stimulus dimension is relevant to visual perception, there should be a lawful relationship between input and output, namely, between stimulus and response. These stimulus-response functions characterize the system in mathematical terms (*R* = *f*(*S*)), and they serve as empirical target for theoretical and computational models of perception. This is the psychophysicist’s approach to “peer into” the black box.

**Figure 1.**
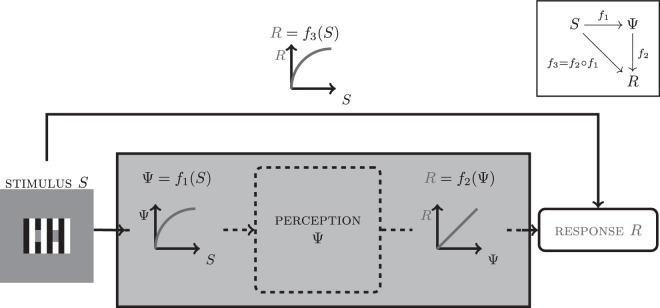
Relationship between stimulus and response for perceptual appearance measures. A stimulus varies along a physical dimension of interest (*S*), e.g., luminance, and observers have a corresponding perceptual experience (Ψ(*S*)), e.g., brightness. Different values of *S* produce different values of (Ψ(*S*)). We refer to this mapping between physical and perceptual quantities as *perceptual encoding*. In a psychophysical experiment, observers are presented with varying *S* and express their perceptual experiences (Ψ(*S*)) by a certain response *R*, such as matching the luminance of a probe stimulus so that it looks like the target. This observable mapping can be characterized by the function *R* = *f*_3_(*S*). Internally, we assume a second mapping, the *perceptual decoding* function which assigns responses to perceptual magnitudes *R* = *f*_2_(Ψ). *f*_1_ and *f*_2_ are happening in a black box called participant. The stimulus response function *f*_3_ = *f*_2_○*f*_1_ is a composition of the encoding and decoding function (inset). To estimate the perceptual encoding function from the stimulus response function, one needs to make assumptions about the perceptual decoding function and vice versa (after Gescheider, 1997).

The actual target of perception research, however, are perceptual processes (Ψ(*S*) in Figure 1), which we infer from observable behavior (verbal reports or button presses). The psychophysical characterization of perception in terms of observable responses (*R* = *f*(*S*)) involves two putative processes (Figure 1; adapted from Gescheider, 1997, Figure 12.7). The perceptual process captures the translation of stimulus variations into perceptual magnitudes (Ψ = *f*_1_(*S*)). It has been called transducer function in the study of near-threshold vision (e.g., Kingdom & Prins, 2016) and stimulus transformation function or psychophysical law in the study of supra-threshold vision (Gescheider, 1997; Gescheider, 1988). We refer to it as perceptual encoding. The second process involves the translation of a perceptual magnitude into a behavioral response (*R* = *f*_2_(Ψ)). It has been called response transformation function, sensory-response law (Gescheider, 1988; Gescheider, 1997), or readout. We refer to it as perceptual decoding. The overall stimulus-response function is thus a composition of perceptual encoding and decoding (*R* = *f*_2_○*f*_1_ see Figure 1).

### Encoding and decoding in near-threshold vision

In the study of near-threshold perception (i.e., detection or discrimination), it is relatively straightforward to model encoding and decoding processes separately (e.g., Graham, 2011, for review). In a discrimination experiment, an observer is presented with two stimuli and asked to choose the stimulus of higher intensity. Signal detection theory (Green & Swets, 1966) assumes that each of the two stimuli evokes a response on the sensory axis. The mapping between stimulus and internal response is noisy and hence varies slightly from trial to trial (perceptual encoding). The perceptual decoding process, i.e., the decision, is then conveniently modelled as the difference between the perceptual magnitudes in the presence of noise. If the difference is larger than some criterion the observer chooses one behavioral response option; if not, they choose the other. This decoding process transforms the quantitative difference in perceived magnitude between the two stimuli into a binary response in an individual trial. However, using many repetitions for the same physical stimulus differences one can infer their perceptual distance from the frequency of correct discriminations.

### Encoding and decoding in supra-threshold vision

In the study of supra-threshold perception, such as perceived size, color, or lightness, it has been more difficult to disentangle encoding and decoding processes. Appearance judgments involve an absolute rather than a relative assessment of intensity, and hence presumably involve anchoring and scaling mechanisms (e.g., Kingdom, 2011, for review). Such mechanisms have not been incorporated in computational models of appearance yet. In the domain of lightness perception, which we are interested in here, existing computational models predict only the direction of a lightness difference (Betz, Shapley, Wichmann, & Maertens, 2015b), or infer the magnitude of perceived differences from the model output to different stimuli relative to one another (Robinson, Hammon, & de Sa, 2007).

Empirically, appearance has also been assessed traditionally with absolute rather than relative judgments. Stevens’ (1956) magnitude estimation required observers to assign numbers to variations in stimulus intensity. This is a difficult task, because it relies on observers’ numerical literacy. Apart from that, the derived scales cannot be taken as direct estimates of the encoding function, because it is unclear how observers map the perceptual magnitudes to numerical responses. This would require assumptions about the (linearity of the) decoding functions (Gescheider 1988; Gescheider, 1997). In some cases, these (implicit) assumptions may hold true, and scales derived from magnitude estimation then should agree with scales derived using methods that make these assumptions explicit (see, for example, Devinck & Knoblauch, 2023).

**Figure 2.**
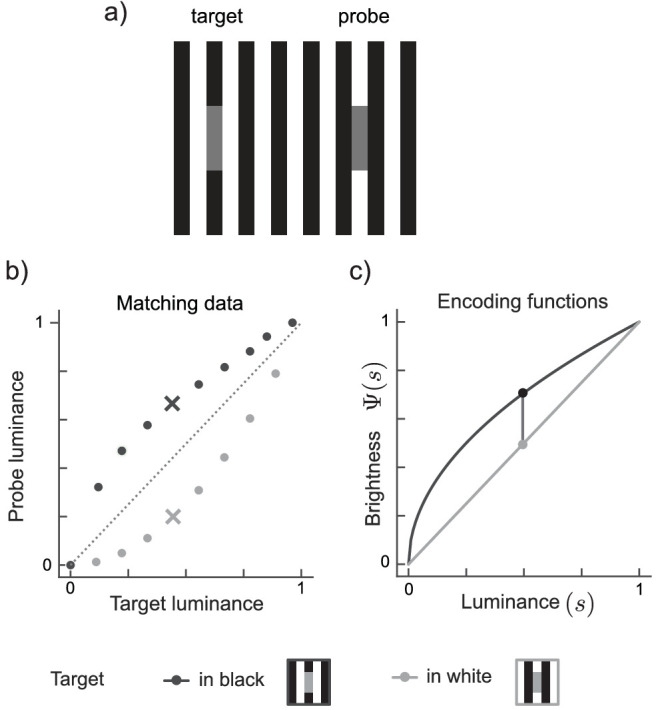
Brightness matching experiment with White's (1979) stimulus. This stimulus (a) produces a brightness difference between equiluminant gray patches embedded in the black (target) and white phase (probe) of a squarewave grating. To match the target’s brightness, observers adjust the probe luminance to be higher than that of the target (dark cross in b). The difference in physical luminance required for a brightness match varies systematically with target luminance. Measurements across the range of target luminances trace out a transfer function (dark gray markers). By swapping the positions of target and probe the complementary transfer function is obtained (light gray markers). (c) Putative encoding functions describing the luminance to brightness mapping in White’s effect. The vertical line, which connects circular markers on both transfer functions, quantifies the perceived difference between equiluminant target and probe.

The predominant experimental paradigm to assess perceptual appearance is matching (Fechner's, 1889, original method of average errors). Matching is a straightforward task where observers physically adjust a probe so as to maximally match some aspect of a given target. In lightness matching (Figure 2), observers adjust the luminance of the probe so that it looks equally light as the target of a given luminance. Matching tasks probe observers’ percepts, i.e., lightness, in the physical “currency” of the stimulus, i.e., luminance. Therefore, they do not have to make explicit assumptions about the internal representation of the perceptual magnitude under study. As long as an experimenter is solely interested in quantifying one perceptual phenomenon relative to another one, matching will do the job just fine. If, however, one is interested in the representation of the perceptual magnitude under study, namely, the perceptual encoding function, we argue that matching data are inapt to estimate these functions.

In this paper, we use the study of lightness perception^1^ to elucidate the implicit assumptions about encoding and decoding processes in matching tasks. We show analytically and in simulation that data from matching tasks do not sufficiently constrain perceptual encoding functions because there exist an infinite number of pairs of encoding functions that generate the same matching data. We then use simulation to demonstrate that maximum likelihood conjoint measurement (Ho, Landy, & Maloney, 2008; Knoblauch & Maloney, 2012) does an excellent job of recovering the shape of ground truth encoding functions from data that were generated with these very functions. Finally, we measure perceptual scales and matching data for White’s effect (White, 1979), and show that the matching data can be predicted from the estimated encoding functions, down to individual differences.

## Encoding and decoding in matching

Figure 2a illustrates a lightness matching task for White's (1979) stimulus. Target and probe have equal luminance, yet the target which is presented “in” the black phase looks lighter than the probe which is presented “in” the white phase. To match the lighter appearance of the target, the observer adjusts the probe to a higher physical luminance than that of the target (Figure 2b, dark symbols). Perceived target lightness is quantified by the luminance of the probe. The deviation of the data from the unity line (black dots relative to dotted diagonal in Figure 2b) quantifies the effect that targets in the black phase appear lighter than targets in the white phase. When the roles of probe and target are reversed, the probe is adjusted to a lower physical luminance (Figure 2b, light symbols). Such mutual matches reflect the effect of context on both probe and target. Alternatively, experimenters might perform asymmetric matching where the probe is embedded in an external field outside the stimulus. Mutual and asymmetric matches might yield slightly different effect sizes, yet both gauge the underlying perceptual representation of the stimulus by probing and expressing a perceptual quantity (lightness) in units of a physical quantity (luminance).

To explain that targets of the same luminance differ in perceived lightness, it is assumed that the mapping from luminance to lightness is different for targets in the black and in the white phase of White's (1979) stimulus. In other words, the lightness encoding functions (or lightness transfer functions, e.g., Adelson, 2000; Maertens & Shapley, 2013; Zeiner & Maertens, 2014) differ between contexts (Figure 2c). The perceived lightness difference for two equiluminant targets is captured by the *vertical* distance between the two encoding functions (vertical line in Figure 2c).

**Figure 3.**
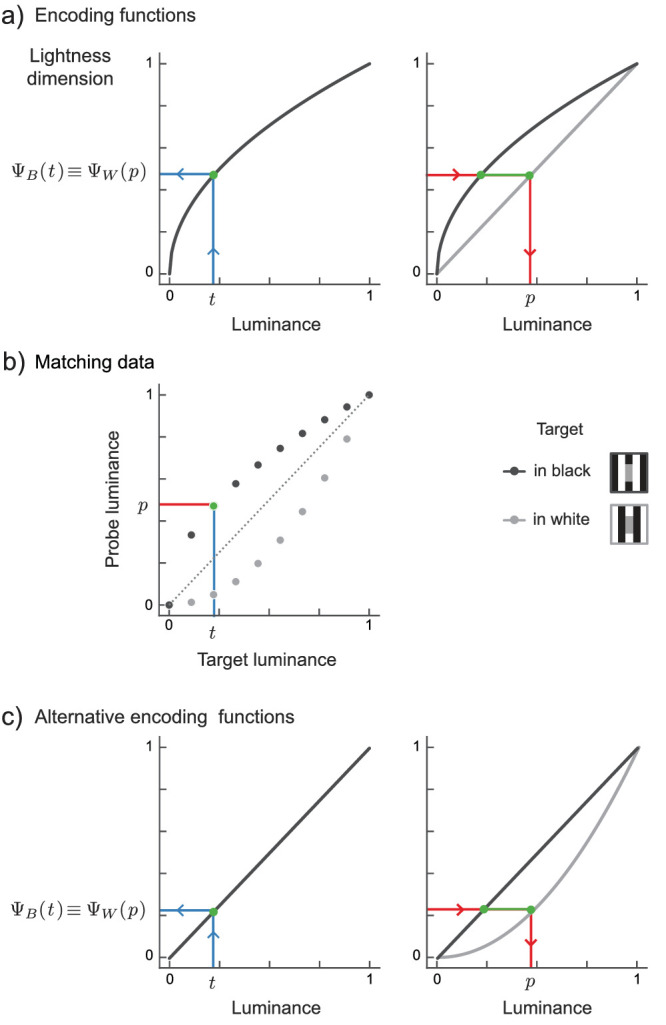
Encoding and decoding in lightness matching. (a) Hypothetical encoding functions that map luminance (x-axis) to perceived lightness (y-axis). A target of a given luminance (*t*) is presented in the black phase of White’s stimulus. The encoding function for the black phase context (Ψ_*B*_) specifies the lightness value (Ψ_*B*_(*t*)) assigned to that luminance (blue arrow). To produce a match, the observer adjusts the probe luminance (*p*, red-line) such that the perceived lightness of the target (in black) and the probe (in white) are identical (Ψ_*B*_(*t*) ≡ Ψ_*W*_(*p*)). The probe luminance is found by inversely reading out the encoding function in the white phase (Ψ_*W*_). (b) Matching data (markers) for different luminances obtained from the procedure described in (a). (c) Alternative encoding functions that produce the same matching data as in (b).

Figure 3a depicts the putative encoding and decoding processes in a lightness matching task. The observer is presented with a target of a given luminance (*t*) in the black phase of White’s stimulus. Using the target encoding function (black curve in Figure 3a) a lightness value (Ψ_*B*_(*t*)) is assigned to the luminance of the target. To produce a match, the observer adjusts the probe luminance (*p*, Figure 3a red line) such that the perceived lightness of the target (in black) and the probe (in white) are identical (Ψ_*B*_(*t*) ≡ Ψ_*W*_(*p*)). The probe luminance (*p*) is found by inversely reading out the encoding function in the white phase (Ψ_*W*_).

Matching provides the *horizontal* difference between target and probe encoding functions in units of luminance for the same ordinate value (Ψ(*t*) ≡ Ψ(*p*), green line in Figure 3a). It does not provide the *vertical* difference between the encoding functions which captures the perceived lightness difference for equiliminant targets.

To quantify the horizontal differences between both functions across the entire stimulus range, one can collect matches for a variety of target luminances, *t*_1_, *t*_2_, .... Unfortunately, as illustrated in Figure 3c, different pairs of encoding functions can produce the same set of matching data. In fact, any pair of encoding functions for which the horizontal distance at each ordinate position is identical, will produce identical matching data. For functions from the power family this holds true for all pairs with the same ratio of their exponents (see Appendix A for analytical derivation). Thus matching data do not sufficiently constrain the putative encoding functions, and hence do not allow to characterize the perceptual magnitudes (Ψ_*B*_(*t*) and Ψ_*W*_(*p*)). In realistic experimental settings this under-determinancy is exacerbated by two factors: noise in sensory events and a selective sampling of matches around a point of maximum difference. We illustrate these two factors in simulation (see Appendix B).

In what follows, we explore an alternative method for estimating encoding functions. MLCM (Ho et al., 2008; Knoblauch & Maloney, 2012) is a difference scaling procedure that yields perceptual scales from difference judgments. The method makes explicit assumptions about noise in perceptual judgments and about the decoding process (*f*_2_). Therefore, the measured perceptual scales should be empirical estimates of the underlying encoding functions. We evaluate this claim in simulation and experiment.

## Encoding and decoding in MLCM

We simulate an MLCM experiment assuming two encoding functions, one for the target in the white (Ψ_*W*_) and one for the target in the black phase (Ψ_*B*_), as in Figure 2c. The functions are defined as power functions of the form Ψ_*B*_(*s*) = *s*^α^ and Ψ_*W*_(*s*) = *s*^β^ where the exponents α, β > 0. In White's (1979) stimulus, targets in the black phase appear brighter than targets in the white phase; therefore, α < β. To test the capability of MLCM to recover the putative encoding functions we use pairs of ground truth encoding functions with different exponents (different shapes), but the same exponent ratio. To cover a wide range of function shapes, we varied α between 0.25 and 2.0, and β between 0.5 and 4.0.

**Figure 4.**
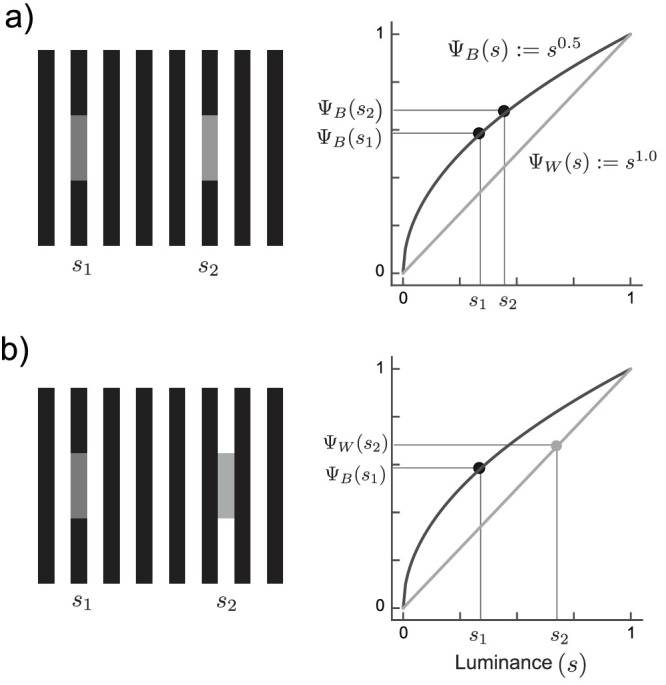
Assumed decoding process for MLCM for two example trials. Two targets *s*_1_ and *s*_2_ evoke perceptual magnitudes Ψ(*s*) thorough their corresponding encoding functions (in black Ψ_*B*_ and in white Ψ_*W*_). These magnitudes along the internal dimension (y-axis) are compared via a difference rule (see formula in text): if the difference is negative, the first stimulus is chosen; otherwise, the second. Responses are collected in this way for all possible pairwise comparisons, within-context (a) and across context (b).

In each trial a (simulated) observer is presented with two targets (Figure 4). Each target is shown in one of two contexts (*c*_1_ and *c*_2_) with a particular luminance (*s*_1_ and *s*_2_). The contexts can be identical (*c*_1_ = *c*_2_ both targets in the black or both in the white phase, as in Figure 4a) or different (*c*_1_ ≠ *c*_2_ one target in the black and one in the white phase, as in Figure 4b). The simulated observer derives two perceptual magnitudes $\Psi_{c_{1}}\left(s_{1}\right)$ and $\Psi_{c_{2}}\left(s_{2}\right)$ which correspond to the luminance value on the respective encoding function (Ψ_*B*_(*s*_1_) for targets presented in the black phase and Ψ_*W*_(*s*_1_) otherwise). To decide which target is brighter, a decision variable δ is computed as the difference between the two perceptual magnitudes:

$$\begin{array}{c} \delta =\Psi_{c_{2}}\left(s_{2}\right)-\Psi_{c_{1}}\left(s_{1}\right)+\varepsilon \end{array}$$

The decision variable δ is assumed to be perturbed by Gaussian noise with zero mean and fixed variance (ϵ ∼ *N*(0, σ^2^)). The simulated observer performs a binary decision. If δ < 0, they choose the first stimulus; if not, the second. We simulated noise with σ values of 0.03, 0.06, and 0.15. These values correspond with the minimum, average, and maximum noise observed in a previous experiment (Aguilar & Maertens, 2020).

The critical assumptions, which allow MLCM to estimate perceptual scales (encoding functions), are the following: (1) variations in stimulus intensity from both contexts are mapped onto *a single internal dimension* (lightness), (2) variability on the internal dimension (noise) is fixed across the scale, (3) the functions that map luminance to lightness are different between the two contexts, and (4) some comparisons must be difficult, so that in some trials δ is small (see Appendix C for an explanation). Although assumptions one to three are a priori assumptions, assumption four depends on the domain under study, that is, the shape of the encoding function, the amount of noise, and the chosen stimulus levels. We simulated the experiment with different parameters of the ground truth functions and different noise levels, and used ten stimulus levels across the possible contrast range.

### Design

We varied target luminance and target placement. We tested 10 luminance values spaced linearly between 0.1 and 0.9, and 2 target positions, in the black or in the white phase of the grating. This results in a set of 20 possible stimuli (10 luminances × 2 placements). MLCM requires the (simulated) observer to “see” and compare all possible stimulus pairs, that is, (20 × (20 − 1))/2 = 190. These judgments form the basis for the scale estimation. Each stimulus was repeated 15 times, resulting in a total of 2,850 trials per simulation. The simulated data were fed into the MLCM estimation routine to estimate (simulated) perceptual scales. We used the MLCM implementation in (R Core Team, 2021; Knoblauch, Maloney, & Aguilar, 2022). We used bootstrap procedures to estimate confidence intervals and evaluate goodness of fit (see Aguilar & Maertens, 2020; Aguilar & Maertens, 2022 for details).

### Evaluation

We repeat the simulation procedure 1,000 times to get estimates of perceptual scale averages and their 95% confidence intervals. To quantify how accurately MLCM can estimate encoding functions, we calculate the root-mean squared error (RMSE) between the obtained perceptual scale $\hat{\Psi }\left(s\right)$ and its ground-truth counterpart (Ψ(*s*)) with *N* = 10 luminance values.

$$\begin{array}{ccc} RMSE & \;= & \sqrt{\frac{1}{N}\sum_{i=1}^{N}{\left[\Psi_{B}\left(s_{i}\right)-{\hat{\Psi }}_{B}\left(s_{i}\right)\right]}^{2}}\\ & & +\sqrt{\frac{1}{N}\sum_{i=1}^{N}{\left[\Psi_{W}\left(s_{i}\right)-{\hat{\Psi }}_{W}\left(s_{i}\right)\right]}^{2}} \end{array}$$

The average error was calculated across simulations. The range of Ψ(*s*) is from 0 to 1 and we interpret this value as average error in percent.

### MLCM estimates encoding functions

**Figure 5.**
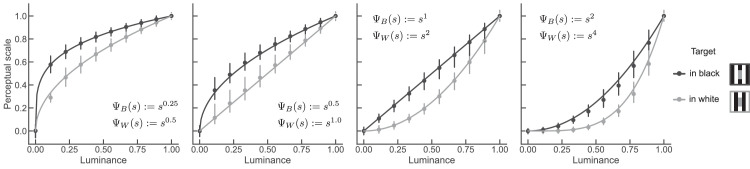
MLCM scales and ground truth functions. Each panel depicts a pair of ground-truth encoding functions (continuous lines) and the average perceptual scales obtained from MLCM (dot markers). Error bars depicts the 95% confidence interval calculated across 1,000 simulation runs. The simulated noise level was 6% (σ = 0.06).

Figure 5 shows the simulated MLCM results for four pairs of encoding functions. It is evident that the estimated perceptual scales (markers in Figure 5) are close to the ground-truth functions for all function shapes. Small deviations occur in the function’s most nonlinear range (Figure 5 left- and rightmost panels). The RMSE, which quantifies the amount of deviation from ground truth, ranged from 2.7% to 3.7% (mid-right and rightmost panels in Figure 5, respectively).

We explored the effect of noise magnitude on estimation accuracy. We repeated the simulations for smaller and larger noise values (σ = 0.03 and 0.15). For larger noise, the perceptual scales were still in close agreement with the ground truth functions (RMSE ranges from 3.0% and 3.4%) (Figure A5). For lower noise, the estimated scales deviated more from the ground truth functions, in particular in the nonlinear regime (RMSE ranges from 3.4% and 5.8%) (Figure A5 uppermost panel). When there is little noise, judgments are almost deterministic (frequency of judging one stimulus as brighter than the other is 0 or 1). Such data introduce bias in the statistical model underlying MLCM, because they carry too little information about the separation of the stimuli on the internal axis. This problem is known as “complete separation” in the logistic regression literature. Our simulation results indicate that for the chosen functions shapes, realistic noise levels and the chosen stimulus spacing, MLCM can recover the shape of the encoding functions.

**Figure 6.**
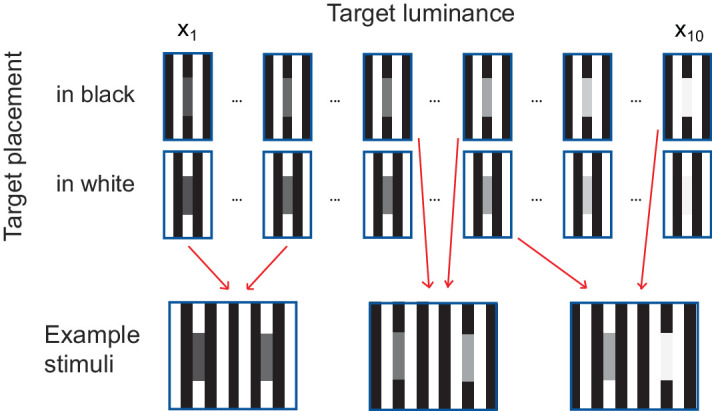
Stimuli construction. Targets varied along 10 different luminance values and position, either “in” the black or “in” the white phase of the grating. Stimuli were constructed by producing all possible paired combinations of these 20 different target types, for a total of 190 stimuli.

**Figure 7.**
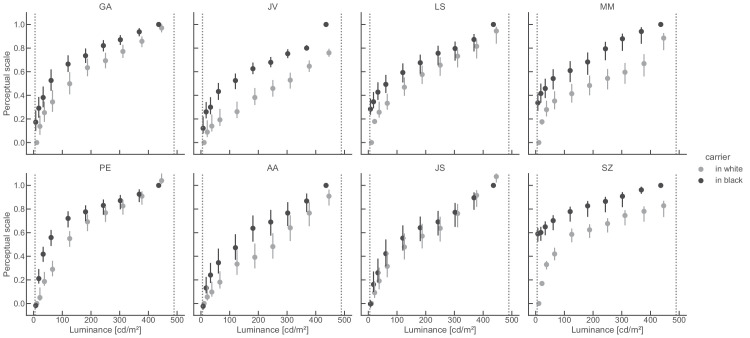
Perceptual scales for White's (1979) stimulus obtained with MLCM. Each panel shows the scales from one participant for targets placed in the white and the black phases of the grating (color). Participants in the upper row were experts, participants in lower row were naive. Perceptual scales were estimated using the “saturated” MLCM model. Error bars depict 95% confidence intervals. Dashed vertical lines indicate the luminance of the black and white grating phases in the stimulus.

**Figure 8.**
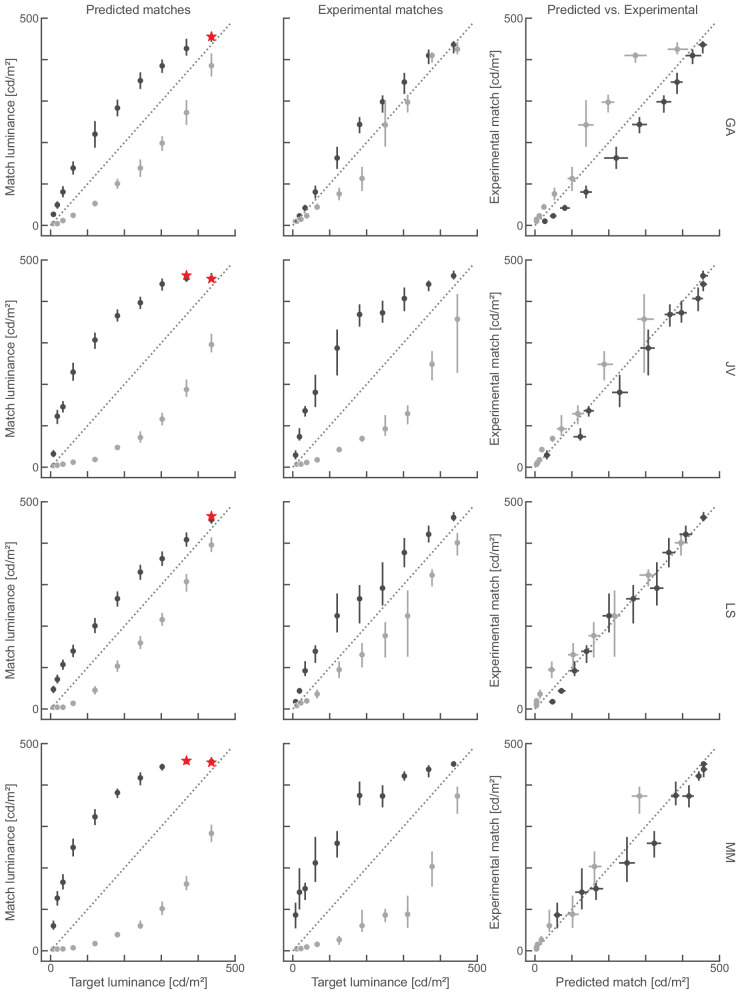
Scales can predict matching data. (Left) Matches derived from perceptual scales following the decoding as outlined in Figure 3. (Middle) Matching data collected in the experiment, for four expert participants (rows). (Right) Replots of the data from left and middle to allow a direct comparison, where perfect predictions would produce the identity line (dashed diagonal). Red stars in the left indicate an incomplete match predicted by the decoding. Errorbars indicate mean 95% confidence intervals.

**Figure 9.**
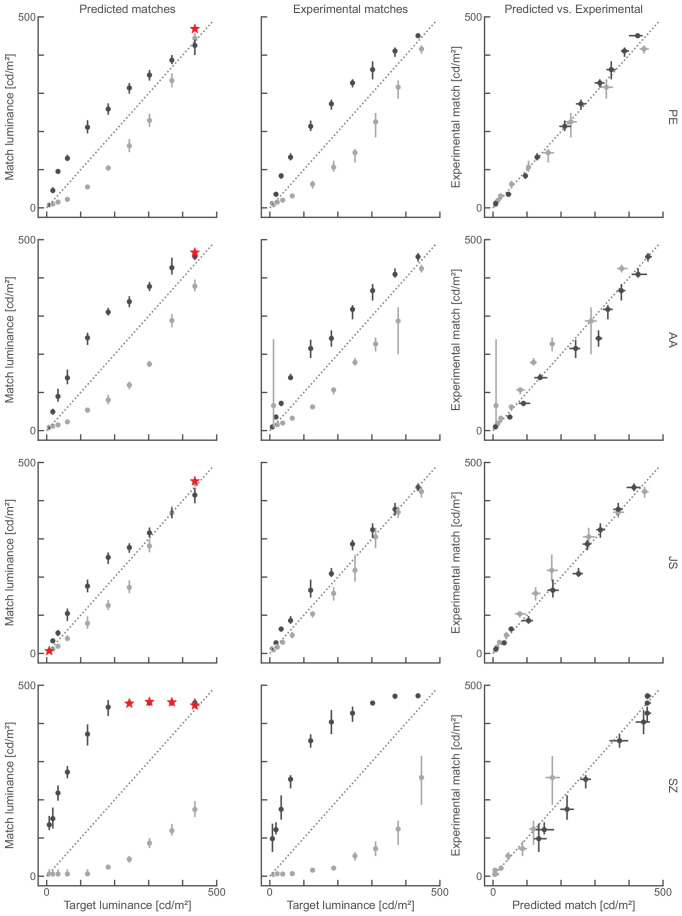
Scales can predict matching data (cont.). Same as Figure 8 but for four naive participants.

**Figure A1.**
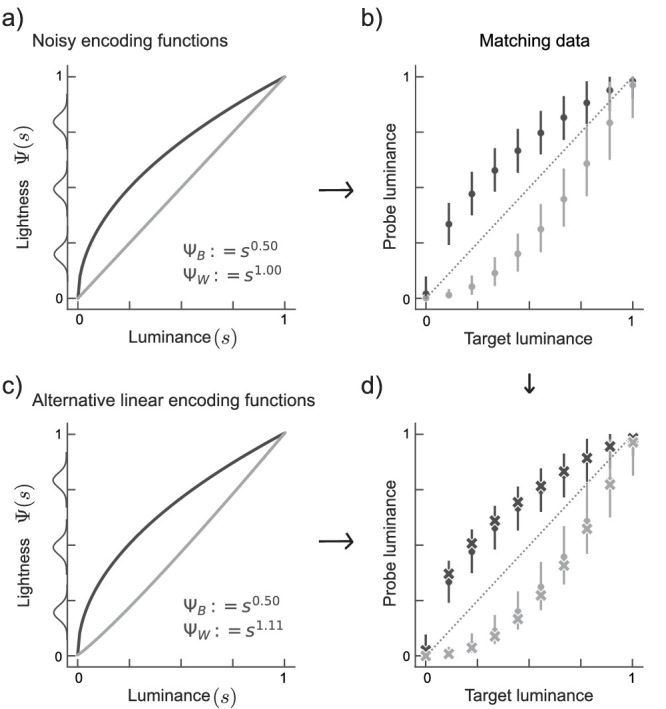
Simulation of matching data in the presence of noise. (a) Non-linear encoding functions used as ground-truth for simulations. Additive Gaussian noise (bell curve icons on the y-axis) is assumed to perturb the lightness representation (Ψ(*s*)). (b) Matching data generated from the encoding functions in a (see text). Markers and error bars depict the mean and the 95% confidence interval across 1000 simulations, respectively. (c) Alternative pair of encoding functions with different exponent ratio. (d) Mean matching data (crosses) generated from the new encoding functions in c, superimposed on 95% confidence intervals (bars) and means (dots) generated from the encoding functions in a.

**Figure A2.**
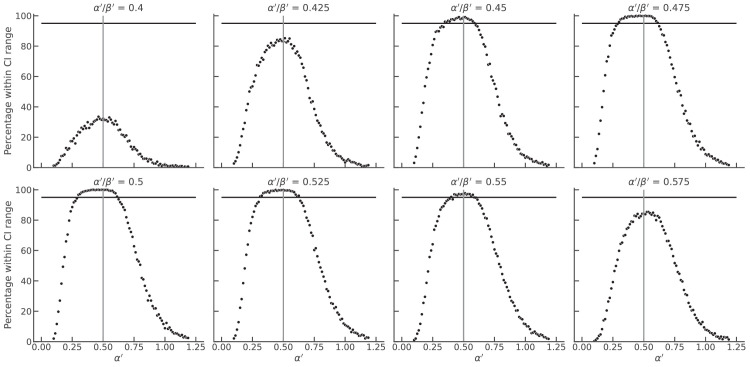
Comparison between simulated matching dataset obtained from original encoding functions Ψ_*B*_ ≔ *s*^0.5^ and Ψ_*B*_ ≔ *s*^1.0^, and matching data from alternative functions of varying shapes. The alternative encoding functions for targets in the black and the white phase varied in their exponent ratio (α′/β′, panels) and α′ (x-axis) The exponent β′ resulted from the other two variables. The vertical line indicates the original exponent ratio of α/β = 0.5. The y-axis depicts the percentage of simulations for which matching data fell inside the 95% confidence interval (as in Figure A1D). The cases above 95 % (horizontal line) were considered as experimentally indistinguishable.

**Figure A3.**
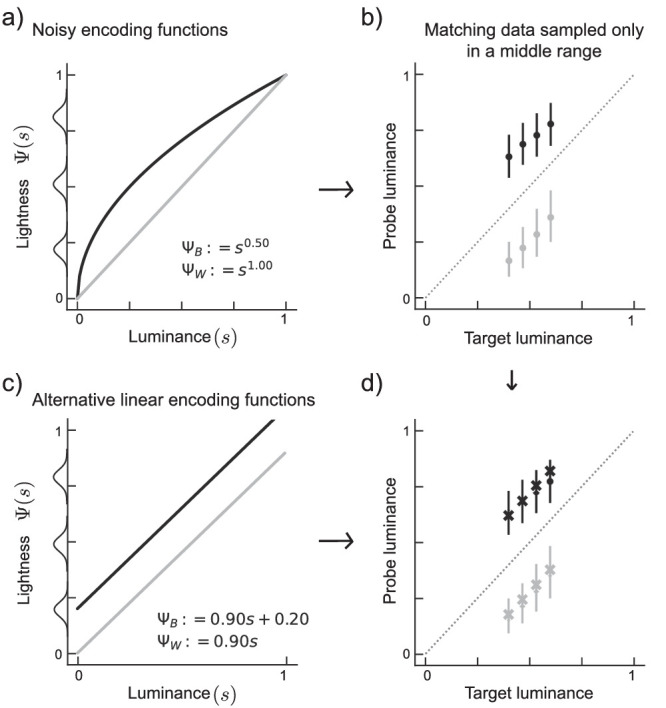
Simulation of matching data with restricted luminance range. Panels are organized as in Figure A1. (b) Matching data are generated from original noisy encoding functions (a) for luminances from 0.4 to 0.6. Matching data (crosses in d) generated from linear encoding functions (c) are compatible with the original data (error bars in d reproduced from b).

**Figure A4.**
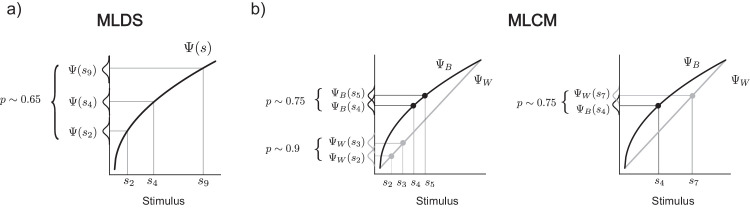
Interval comparisons in MLDS and MLCM. In MLDS an observer compares perceptual intervals of triads (as depicted in a) or quadruples (not shown). In MLCM observers perform paired comparisons (b). To establish a metric scale or scales, all paired comparisons are taken together in the same model across trials. The interval comparison is thus not done within a single trial but by subsuming performance for different interval comparisons across trials. In panel b, one interval within the same context (left side, black markers, Ψ_*B*_(*s*_5_) − Ψ_*B*_(*s*_4_)) is equivalent to another interval across contexts (right side, black and gray markers, Ψ_*W*_(*s*_7_) − Ψ_*B*_(*s*_4_)), because their response frequencies are the same (*p* ∼ 0.75).

**Figure A5.**
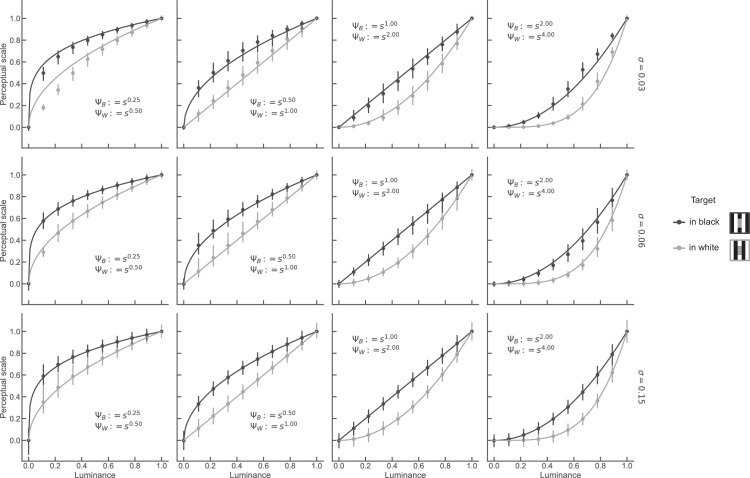
Results of simulated MLCM experiment for varying function shapes (columns) and amounts of noise (rows, σ). Same format as Figure 5 in main text.

## Experimental method and results

In the following experiment, we use MLCM to measure perceptual scales for White's (1979) effect. We also measure perceptual matches in White's (1979) effect for the same participants. We compare the empirical matches to matches predicted from the perceptual scales.

### Participants

Four expert participants (the three authors and one affiliate) and four naive participants participated in the experiment. Naive participants were financially compensated for their participation (€12/hour). All participants had normal or corrected-to-normal vision. One participant (GA) has deuteranomalous color vision.

### Apparatus

Stimuli were presented on a 21-inch Siemens SMM2106 LS grayscale monitor driven by a Datapixx device (Vpixx Technologies, Inc., Saint-Bruno, QC, Canada) and custom presentation software (HRL, https://github.com/computational-psychology/hrl). The apparatus allows a luminance depth resolution of 16-bit, with a spatial resolution of 1,024 × 768 pixels (400 × 300 mm) and at a 130-Hz refresh rate. Monitor calibration and luminance measurement was conducted using a Minolta LS-100 photometer (Konica Minolta, Tokyo, Japan). Participants viewed the stimuli from a chinrest positioned approximately 76 cm away, such that 1° visual angle corresponded with 34 pixels. Participants’ answers were recorded with a ResponsePixx button-box (Vpixx Technologies, Inc.).

### Stimuli

Stimuli were versions of White's (1979) stimulus, based on Robinson et al. (2007) WE_thin version, and created using a pre-release version of stimupy (Schmittwilken, Maertens, & Vincent, 2023). The stimuli consisted of two gray targets patches embedded in a high contrast square-wave grating (Figure 2a). The square-wave grating spanned 16° × 12° (width × height), with a frequency of 0.5 cycles per degree, so that the stimuli contained exactly 8 full cycles (16 black and white bars). The minimum luminance, corresponding to the black phase, was 5.25 cdm^−2^, and the maximum luminance, corresponding with white phase, was 490 cdm^−2^, producing a Michelson contrast of 0.98. The grating was centered on a neutral gray background of 95 cdm^−2^.

Two target patches were embedded in the grating. Their placement varied from trial to trial according to the design (see below). The phase of the grating was randomized, that is, starting with black or white, and the targets were randomly placed either on phases 4 and 12, or on phases 5 and 13 of the grating (counting from left to right). Target patches spanned 4° vertically, and were vertically centered in the middle of the grating. We tested 10 different target luminances which were nominally identical for both target placements, 1.0%, 3.5%, 7%, 13%, 26%, 39%, 52%, 64%, 77%, 90% of maximum monitor luminance. In practice, the luminances differed slightly for targets placed in the black or white phase of the grating because of monitor inhomogeneities. Targets in the black phase were 7.6, 18, 33, 61, 120, 181, 243, 302, 368 and 436 cdm^−2^, and targets in the white phase were 11, 22, 38, 66, 126, 188, 250, 311, 377, and 446 cdm^−2^. These actual luminances were measured at the target positions with the full stimulus on the display. Thus, the reported values match what participants saw during the experiment.

### MLCM procedure

The experimental design was the same as in the simulations. There were 20 possible target types (10 luminance values × 2 placements), resulting in a total of 190 unique stimulus pairs. Targets were embedded in the grating and according to the design placed in black phases, in white phases, or one in a black and one in a white phase (see Figure 6 for examples).

For each trial, a stimulus was presented and the participant indicated which of the two targets appeared brighter by pressing the left or right button on a response box. The stimulus was shown until the participant pressed a button. Trials were organized in blocks and each block contained all 190 possible stimuli. Participants completed 15 blocks over the course of 3 sessions, for a total of 2,850 trials per participant (the same amount as in the simulations). The sequence of trials within each block was random. Each block lasted on average 4 minutes. Participants were free to take breaks between blocks.

Perceptual scales were estimated using MLCM following the procedure described for the simulations (see the section on Encoding and decoding in MLCM).

### Mutual matching procedure

Participants also completed a mutual matching task with the same stimuli as in the MLCM task. For this, task participants were presented with a target placed in one phase of the grating and were asked to adjust the probe placed in the other phase of the grating. Observers only performed matches where target and probe were presented in different phases of the grating. They were instructed to adjust the probe so as to match the target’s brightness. They could use two buttons for coarse and two buttons for fine adjustments. A fifth button was used to indicate that a satisfactory match had been reached and this triggered the presentation of the next trial. No time limit was imposed. In a single block, each target type (10 luminance values × 2 positions) was repeated twice, once on the left target location and once on the right. Participants completed 3 blocks (1 per session) of this task, resulting in a total of 120 trials per participant.

## Results

### Perceptual scales

Figure 7 shows the perceptual scales for each participant. By default, MLCM anchors the scale minimum (0) at one (arbitrary) stimulus level. The maximum scale value reflects the noise estimated for that participant. The higher the scale value the smaller the estimated noise. Noise estimates ranged from 0.03 to 0.07 across participants, with an average of 0.05. To be able to compare scales between participants, we assigned the lowest target luminance for targets in the white phase as the minimum (scale value of 0), and we divided all scale values per participant by the maximum value of that participant. This results in a maximum scale value of 1.

Perceptual scales were monotonically increasing nonlinear functions of target luminance. Their shape and amount of nonlinearity varied between participants, and also depended on the location of the target. The scale values for targets in the black phase (black markers in Figure 7) were higher than for targets in the white phase for almost all data points. This effect was more pronounced for some participants than for others and is consistent with the direction of White's (1979) effect. Scales from participants JV, MM, and SZ show a large difference between the two scales, whereas participant JS showed almost no difference. As we will show below this inter-observer variability seems to reflect idiosyncratic aspects of how participants perceive the stimulus, because these differences were reproduced in the matching task.

We performed likelihood ratio tests on individual participants’ data to determine which statistical model in MLCM fits the data better (either the “independent,” “additive,” or “saturated” model, which vary in the degrees of freedom; see Knoblauch & Maloney, 2012 for details). Across participants, the most general, “saturated,” model accounted best for the data, and revealed that perceptual scales were not just related to each other by a vertical shift of fixed amount. Instead, the target placement affected the mapping of luminance to lightness in different ways for different target luminances.

### Predicting empirical matches from empirical scales

If we assume that perceptual scales are valid estimates of perceptual encoding functions, and that matching relies on ‘readout’ from these encoding functions (Figure 3; c.f. Aguilar & Maertens, 2020), then we should be able to predict the empirical matches from the empirical perceptual scales.

Figures 8 and 9 show matches derived from perceptual scales next to matching data measured in a mutal matching task for each individual participant. Here the perceptual effect that targets in the black phases look lighter than targets in the white phases is expressed by the deviation of the data points from the identity line. There is substantial variability between participants; however, empirical and predicted matches from the same participant are consistent. For example, naive participant JS shows almost no effect of target location on target brightness, and their matches almost line up on the identity line. That pattern is also predicted from the perceptual scales. SZ on the other hand shows a pronounced effect of target location on target brightness and that pattern of matches is also predicted from their perceptual scales. To better compare the data we plot the empirical matches against the predicted matches (right panels in Figures 8 and 9). Here the identity line indicates perfect prediction. Apart from one participant (GA) there is a close correspondence between empirical matches and those predicted from perceptual scales. However, there is one noteable difference between empirical and predicted matches: empirical matches were less consistent than the predictions as indicated by their wider 95 % confidence intervals (Figures 8 and 9).

## Discussion

### Main findings

We estimated perceptual scales of brightness as a function of target luminance for both targets in White's (1979) stimulus using MLCM (Ho et al., 2008; Knoblauch & Maloney, 2012). For all participants, these scales were nonlinear, and the scale for the target in black was always above the scale for the target in white. This is in line with White's (1979) effect that targets in black are perceived lighter than equiluminant targets in white.

### Shapes of brightness scales

The perceptual scales had well-defined shapes, although there was individual variation. The scale for the target in black was a compressive nonlinearity. For most participants the scale for the target in white had a more pronounced *S*-shape: approximately linear at intermediate values and accelerating towards the ends of the luminance range. Consequently, the scales approach each other at the low and high ends of the range, and bulge away from each other for intermediate luminance values. This shape difference suggests that two isoluminant targets are perceived maximally different at intermediate luminance values, and the magnitude of White's (1979) effect decreases towards the extremes of the luminance range. Appearance matches are often gathered for intermediate target luminance values, and not across the whole range of luminances spanned by the surround context. This makes it harder to compare the shapes of the scales reported here to previously reported measurements of White's (1979) effect. Vincent (2017) reported similar variation in effect magnitude as a function of target luminance in a matching paradigm. Rather than varying the target luminance Lin, Chen, and Chien (2010) varied the contrast of the grating while keeping target luminance constant. They found match contrast decreasing with increasing surround contrast, which could be in line with the same overall shapes of brightness scales described here: lower contrast surrounds would compress the domain of encoding functions, reducing the intermediate range of luminance values where effect magnitude is maximal. The maximal effect magnitude at intermediate values may make a matching task and thus data collection easier, which in turn could be a practical reason why previous measurements have focused here.

The shapes of the scales also bear resemblance to those measured by Whittle (1992). In that seminal work, participants adjusted the luminances of a series of a fixed number of stimuli such that they corresponded to equal brightness steps from black to white. For increment stimuli, brightness was a compressive function of luminance. For decrement stimuli, a similar *S*-shape was reported: steepening of the relationship near both the pedestal luminance as well as the background luminance. Whittle (1992) reported this as the “crispening” effect: an enhancement of brightness differences near background and pedestal luminances, which also appears in brightness discrimination (Whittle, 1986).

The current scales can be said to show crispening as well, if the targets are considered to contrast with (only) the collinear bar that they are “in.” The targets in black are increments and their brightness scale is a compressive function of luminance. The targets in white are decrements and their brightness scale similarly crispens near the white collinear bar as well as near the black minimum luminance. This dominant role for the collinear, but not the flanking, contrast in White's (1979) effect has previously been implied (e.g., Betz et al., 2015a; Betz et al., 2015b; Blakeslee, Padmanabhan, & McCourt, 2016).

### Perceptual scales as estimates for encoding functions

We argue that perceptual scales, such as the lightness scales we derived with MLCM, can be considered estimates of *perceptual encoding* functions. These encoding functions (or estimates thereof) can be thought to underlie both the pairwise comparison data collected in the MLCM experiment, as well as the appearance matches in the matching task. Both our simulations and our empirical data show that brightness encoding functions estimated using MLCM can predict brightness matches from the same participants to within the variability of the matches. These predictions are reliable for each participant and capture the idiosyncratic variations observed in the matches. This good congruence between matches and scales is strong evidence that both tasks tap into the same perceptual encoding mechanisms.

We showed analytically and through simulation that the MLCM-based encoding functions can predict matches, but matches cannot be used to (uniquely) constrain the underlying encoding functions. Depending on noise and measurement range, a wide variety of encoding functions can be consistent with the same set of matches. This is because matching does not separate encoding and decoding processes. Scaling methods such as MLCM separate decoding and encoding, and explicitly define a perceptual decoding strategy. We have used the method in a novel way, because we asked participants to perform pairwise comparisons across a dimension that was categorical (black vs. white context), not metric (e.g., Ho et al., 2008). We have shown in simulation and experiment that perceptual scales can be reliably estimated when there is a sufficient number of non-trivial trials in the set of all comparisons (see Appendix C for explanation). If all comparisons were easy, they would result in a proportion of correct responses of 1. These comparisons are not informative about the size of perceptual intervals. As shown in Figure A4, intermediate performance values are ideal to establish the shape of the perceptual scales. Without the informative trials, estimation would default to linear scales, because the size of different perceptual intervals would not be distinguishable, and hence be estimated to be identical. Whether or not it is possible to estimate perceptual scales for categorical dimensions in other domains of appearance is an empirical question. We recommend to use simulation to trace out the space of function shapes, noise levels and stimulus spacing before collecting data for a particular stimulus.

### Equality vs. difference judgments

Pairwise comparisons are easier than brightness matching. A large number of trials in our MLCM experiment consisted of physical differences between stimuli that were easy to judge (all within context comparions). Only some comparisons between contexts were difficult because they required participants to decide between small differences in brightness. In contrast, the matching task required participants to find a point of perceptual equivalence in every single trial. Instead of judging the direction of a perceptual difference (paired comparison), they minimize a perceptual difference (matching), which is considerably more demanding. Additionally, there might be conditions under which participants may set a match, but contend that the target and probe do not appear identical. The situation may arise when variations along a single physical stimulus dimension lead to changes on more than one perceptual dimension (Logvinenko & Maloney, 2006). For example, for brightness it has been reported that under low luminance (or contrast), the target appears to be seen trough a transparent medium (Ekroll, Faul, & Niederée, 2004). Although those effects occur in both matching and scaling tasks, we think the problem affects matches more than paired comparisons, because it is already an inherently more difficult task.

The difference in inherent task difficulty might explain why the confidence intervals associated with matches predicted from scales are smaller than those for the empirical matches (Figures 8 and 9). Participants’ match luminances (six repeats for a given target luminance) often span a range of brightness values that is way larger than the brightness differences they could discriminate. They consider all these values appropriate matches for the same target brightness. In matching data, this appears as noise or variability, but it may reflect aspects of participants’ perception such as the aformentioned imperfect matching.

Perceptual scales from pairwise comparisons appear less noisy. In part, this is because the stochasticity in a participant’s responses is used to estimate the scales. It is also by experimental design. The stimulus values for MLCM are chosen such that within one context they can be well-discriminated and put in order. This means participants are presented with fewer trials that fall within a given equivalence class. If those equivalence classes reflect some aspect of perception not captured in the brightness difference task, that may not be captured in the brightness scales. Thus, although scaling data are less noisy, they may also miss aspects of perception.

Additionally, the pairwise comparisons in MLCM are faster than the brightness matches. Participants only need seconds to make the single decision on every trial. In contrast, a matching trial can take up to 1 minute, with participants adjusting and readjusting their match. Five blocks of 190 paired comparison trials in the current experiment took about as long as a single block of 40 matching trials. Moreover, there may be room for additional speedup in MLCM tasks. As mentioned, many of the pairwise comparisons are easy trials, which tend to result in deterministic responses (disregarding lapses), and thus carry little information for the scale estimation. Selecting more informative trials could improve the estimation procedure and decrease the total number of trials.

### Anchoring the scales

A potential advantage of matching over scaling, is that participants can explicitly compare to some well-defined standard, such as Munsell papers. This would allow for a more directly interpretatble estimation of the perceptual encoding function, but relies on an (implicitly) assumed shape of the encoding function for the matching stimulus. The external stimulus itself is encoded through some function. There is no fundamental difference between asymmetric and the mutual matching used in the current study. Matching to a well-defined standard instead relies on a known or assumed perceptual encoding function for the matching stimulus. For example, in Munsell matching, the function has perceptually equal spacing by design (Newhall, 1940). In contrast, scaling methods do not require any assumed shape for either encoding function and instead estimate both shapes simultaneously. Pairwise comparisons can also be used to compare a stimulus to a standard, with comparisons for every combination of stimulus level and standard level (across-context), as well as every combination of stimulus levels (within-context), and standard levels (within-context). Scales could then be estimated in the same way as in the current study: one for the stimulus, and one for the standard. The latter can be scaled to the predefined, interpretable units. The comparions between standard levels could even be left out experimentally, instead assumed to be, e.g., perfect noiseless discrimination between standard levels. That is the exact same assumption as Munsell matching, but more explicitly, in our view. We hypothesize that the matches to the standard could be predicted from scales estimated in this way. Thus, scaling methods are more flexible in estimating perceptual encoding functions, and can still be constrained in the same way as matching, but more explicitly so.

### Encoding functions and mechanisms

Perceptual encoding functions describe the relationship between physical stimulus intensities and perceptual mangnitudes. They do not explain this relationship mechanistically, nor do we argue that they represent any one mechanism. A transfer function just describes the relationship between inputs and outputs of a system and aggregates all the mechanisms involved in the system. Thus, a perceptual encoding function may represent different perceptual mechanisms at play. These could have differential effect on different parts of the function. The linear part of the encoding functions might be primarily driven by a mechanism that treats the target as separate object. The crispening at the extremes of the range may be driven by a mechanism that invokes transparency. Our free-floating speculation shows that caution is advised in (over)interpreting a given perceptual encoding function.

We nevertheless argue that encoding functions are a useful step towards developing mechanistic theories of perception. Firstly, because they provide more structured information about perceptual effects than appearance matches, especially when comparing different effects; if we were to compare, for example, several brightness and lightness effects: White's (1979) effect, simultaneous brightness contrast, and brightness assimilation effects. Using a matching paradigm, we can measure and compare the magnitudes of these effects for some luminance value. However, when comparing different effects, it may be difficult to decide which stimulus parameters for the surround contexts provide an equivalent comparison. Should the total image contrast be identical, or the length of contrast borders with the target, or the total area of high *vs.* low luminance context regions, and so on. Differences in effect magnitude measured with matching may result from any of these “trivial” stimulus parameters. While perceptual encoding functions do not solve this problem, they provide more robustness, since we are not comparing single effect magnitudes, but rather a whole relationship between stimulus values and perceptual magnitudes. We may test whether the shape of the functions fundamentally differs between effects, or whether differences are limited to the range, local slope etc. Hence, comparing encoding functions provides more information about the potential relationship between different perceptual effects.

One intriguing challenge, especially in comparing perceptual encoding functions is determining the relevant stimulus parameter(s), especially when these are correlated. For example, here we estimated brightness as a function of target luminance, but varying target luminance also varies the contrast between the target and the collinear bar, and between the target and the flanking bar. Which of these is the relevant parameter, and is that also the relevant parameter in another brightness effect? Which values along this stimulus dimension should we pick to properly sample the perceptual dimension? To estimate the shapes of the perceptual encoding functions, it is important to have good coverage over the whole domain and especially near inflection points of the functions. Because we do not know the shape of these functions beforehand (and they vary between observers), we also do not know which stimulus values will be most informative. The target luminance values for the experiment described here were chosen through pilot testing to better capture the steep slope at low luminances. When measuring and comparing encoding functions for a variety of stimuli, such pilot testing may be necessary to choose relevant stimulus dimensions and a value spacing that provides good coverage for all of them.

## Conclusions

Here we show that data derived with matching paradigms do not uniquely constrain perceptual encoding functions. We used MLCM to derive perceptual scales that are estimates of encoding functions for luminance targets in the White's (1979) stimulus. Scales had clearly defined nonlinear shapes and noteworthy inter-observer variability. We used the scales to predict matches including the interindividual differences. This provides evidence that scales reflect the internal dimension of lightness that is probed by both matching and scaling. We conclude that perceptual scaling data allow us to “peer into” the black box of visual perception, and provide a better target for computational models of perception.

## Footnotes

1 We speak of lightness/brightness perception interchangeably throughout the manuscript although we agree that in White’s stimulus, presented on a monitor, one does not perceive lightness as surface reflectance. In our opinion the distinction is not relevant for the argument we are going to make.

## Appendix A: Analytical derivation of one equivalence class

First, we assume the existence of two distinct encoding functions, one for the target “in” black Ψ_*B*_ ≔ *s* → Ψ_*B*_(*s*) and one for the target “in” white Ψ_*W*_ ≔ *s* → Ψ_*W*_(*s*).

Further, we assume that these two functions map to a single perceptual dimension, (in this case lightness Logvinenko & Maloney, 2006, have shown that this might not be the case). Formally, we assume then that the range of both functions is the same set. In our simulations we have defined it as [0, 1].

Let the target be “in” white and the probe “in” black. During a matching procedure the observer matches internally:

$$\begin{array}{c} \Psi_{B}\left(p\right)=\Psi_{W}\left(t\right). \end{array}$$

What we want is to get a formula for the matching transfer function, that is, the (observable) probe luminance (*p*) as a function of manipulated target luminance (*t*). To do that, we further assume that Ψ_*B*_ is invertible (i.e., one-to-one and onto) and apply it to both sides of the equation

$$\begin{array}{ccc} \Psi_{B}^{-1}\left[\Psi_{B}\left(p\right)\right] & \;= & \Psi_{B}^{-1}\left[\Psi_{W}\left(t\right)\right]\\ p & \;= & \Psi_{B}^{-1}\left[\Psi_{W}\left(t\right)\right]\\ p & \;= & \left(\Psi_{B}^{-1}\circ \Psi_{W}\right)\left(t\right) \end{array}$$

As a result, we obtain a (matching) transfer function relating *t* and *p* as the *composition* of $\Psi_{B}^{-1}$ with Ψ_*W*_. Analogously, when the target is “in” black and the probe “in” white, we obtain the complementary function $p=\left(\Psi_{W}^{-1}\circ \Psi_{B}\right)\left(t\right)$.

For the case when encoding functions are power functions, let Ψ_*W*_ ≔ *x* → *x*^α^ and Ψ_*B*_ ≔ *x* → *x*^β^, with α, β > 0. Let the target be “in” white and the probe “in” black. The inverse of the probe encoding function is $\Psi_{B}^{-1}:=\psi \to \psi^{\frac{1}{\beta }}$. Applying the same logic as above, we have that the probe relates to the target such that:

$$\begin{array}{ccc} p & \;= & \left(\Psi_{B}^{-1}\circ \Psi_{W}\right)\left(t\right)\\ & \;= & {\left[t^{\alpha }\right]}^{\frac{1}{\beta }}\\ & \;= & t^{\frac{\alpha }{\beta }} \end{array}$$

This gives us an analytical formula for a matching transfer function, namely, how the probe luminance depends on the target luminance for the given two encoding power functions. It follows that as long as the ratio between the exponents (α/β) is the same, the resulting observable transfer functions will be identical. Analogously, the same holds for the swapped case of the probe “in” white and the target “in” black, for which the ratio is β/α.

## Appendix B: Simulating matching

We simulate matching data for different pairs of encoding functions, one for the target in the white (Ψ_*W*_) and one for the target in the black phase (Ψ_*B*_). Encoding functions are defined as power functions of the form Ψ_*B*_(*s*) = *s*^α^ and Ψ_*W*_(*s*) = *s*^β^ where the exponents α, β > 0. In White's (1979) stimulus targets in the black phase appear brighter than targets in the white phase, therefore α < β (e.g., Figure A1a).

We use power functions, because 1) they capture the compressive nonlinearities observed in brightness perception, 2) they range between 0 and 1 for (normalized) inputs between 0 and 1, and 3) their inverse is simple to calculate. The logic outlined here applies to other pairs of (nonlinear) functions as long as their horizontal differences are the same. We further model the perceptual representation (Ψ) underlying the brightness match as perturbed by noise (bell curve icons in Figure A1a). For a given target luminance *t*, the perceptual variable (ψ_*t*_) is thus calculated as a sample from a Gaussian random distribution:

$$\begin{array}{c} \psi_{t}∼N\left(\Psi_{T}\left(t\right),\sigma^{2}\right), \end{array}$$

where Ψ_*T*_ is the encoding function for the target (Ψ_*B*_ or Ψ_*W*_, for targets in the black or the white phase, respectively) and the parameter σ = 0.05 denotes the amount of simulated noise.

To readout the probe luminance for the brightness match, the probe encoding function Ψ_*P*_ is inverted to assign a probe luminance (*p*) to perceived target brightness (ψ_*t*_):

$$\begin{array}{c} p=\Psi_{P}^{-1}\left(\psi_{t}\right). \end{array}$$

For simplicity, we do not add noise to the probe readout, and we also do not assume any response bias.

We simulate matches for 10 target luminances linearly spaced between 0 and 1, and sample 5 matches per target. The roles of target and probe are swapped to obtain matching data for targets in the black and in the white phase. This results in a total of 100 trials per simulation (2 targets × 10 luminances × 5 samples). A simulation is repeated 1,000 times for each pair of encoding functions to get an estimate of the mean and the 95% confidence interval (percentile method). We provide Jupyter notebooks (available at https://github.com/computational-psychology/encoding_functions_and_white_stimulus), which run the following simulations and visualize the data for various pairs of encoding functions.

### Matching data in the presence of noise

We illustrate the procedure for encoding functions with exponents α = 0.5 and β = 1.0, in the black and white phase, respectively. Figure A1a shows the ground-truth encoding functions and Figure A1b the matching data generated from these functions in the presence of noise.

We know from the analytical derivation (Appendix A) that any other pair of power functions with the same exponent ratio (in this case α/β = 0.5) will produce the same pattern of matching data. In the presence of noise the number of interchangeable pairs of encoding function will be even bigger.

To numerically estimate the effect of noise on the under-determinacy of the underlying encoding function, we simulate pairs of encoding functions with exponent ratios different from 0.5. We hypothesize that encoding functions with a ratio somewhat smaller or larger than 0.5 will generate matches that are indistinguishable from matches generated from the original functions.

Figure A1c shows an example pair of alternative encoding functions (α/β = 0.50/1.11 = 0.45), and a corresponding simulation of generated matching data (Figure A1d, crosses). The mean matches of the simulated data set (crosses) fall within the 95% confidence interval of the original matches. We take this as evidence that data generated from functions with exponent ratios 0.5 and 0.45 are empirically indistinguishable.

We repeat this simulation logic for exponent ratios ranging from 0.4 to 0.6 (in linear steps of 0.025), with α ranging from 0.1 to 1.2 (in linear steps of 0.0125). The exponent β resulted from these two variables. We found that encoding functions with a wider exponent ratio produced matching data consistent with data from the original encoding functions. Figure A2 shows the percentage of simulations in which matching datapoints fell in the range determined by the original data across 1000 simulations. As above, the cases over 95% are considered ‘in agreement’ with the original data. The following cases were in agreement: for ratio α/β = 0.45: α in the range [0.35, 0.59] and β in [0.78, 1.31]; for ratio α/β = 0.475: α in the range [0.3, 0.61] and β in [0.63, 1.29]; for ratio α/β = 0.50: α in [0.3, 0.61] and β in [0.6, 1.2]; for ratio α/β = 0.525: α in [0.31, 0.63] and β in [0.6, 1.2]; and for ratio α/β = 0.55: α in [0.4, 0.56] and β in [0.73, 1.1].

### Matching data from a restricted range of samples

In actual experiments, matches are often restricted to intermediate values of the stimulus dimension, where the perceptual effect is expected to be strongest. Here we simulate this case. Figure A3a shows the same nonlinear encoding functions as Figure A1a, but now matches are generated only for target luminances in the range between 0.4 and 0.6 (Figure A3b). The simplest type of encoding function that would account for matching data in this restricted range would be a linear one. We repeat the same procedure as for the exploration of exponent ratios and now simulate matching data for a family of linear encoding functions varying slope and intercept (slopes in range 0.8 and 1.0, intercept in range −0.2 and 0.2). Across the tested range linear encoding functions produce matching data, which are indistinguishable from data generated with the original encoding functions (see Figure A3c for an example).

In summary, we find that in realistic experimental scenarios the set of possible encoding functions compatible with a given matching dataset is wide. Thus, matching data alone do not allow to infer the underlying encoding functions.

## Appendix C: MLCM assumptions and scale estimations

In this appendix, we explain in detail the assumptions behind MLCM, and how they allow the method to estimate perceptual scales from paired comparisons. To better understand the logic, we briefly review the general assumptions of both maximum likelihood difference scaling (MLDS) and MLCM, because the methods are closely related to each other.

MLDS and MLCM are methods to estimate perceptual scales based on binary responses to suprathreshold stimulus differences (Knoblauch & Maloney, 2012). MLDS is designed to estimate a *single* perceptual scale. It assumes that variations on a single stimulus dimension *s* map to variations on a *single* perceptual dimension Ψ(*s*). MLCM is designed to estimate *more than one* perceptual scale. It assumes that stimulus appearance along one perceptual dimension is determined by several physical dimensions. In our case we assume that perceived target lightness is affected by the luminance and the context of a target. MLCM estimates perceptual scales which map variations in luminance to variations in perceived lightness, and these scales differ between contexts. To take the internal stochasticity of human judgments into account MLDS and MLCM include a noise parameter in the model. Noise is assumed to be constant across the perceptual dimension and is estimated along with the scale(s).

The noise can be thought to occur at the decision stage, that is, after perceptual magnitudes are compared. This idea is reflected in the decision model of MLDS and MLCM, which is formulated as δ = (Ψ(*s*_3_) − Ψ(*s*_2_)) − (Ψ(*s*_2_) − Ψ(*s*_1_)) + ϵ for an MLDS trial where three stimuli need to be compared. Alternatively, the mapping to the internal dimension itself can be noisy, and hence the same stimulus magnitude would be associated with a distribution of perceptual magnitudes as illustrated by the Gaussian curves on the y-axis in Figure A4. Because noise is assumed to be Gaussian and of equal variance along the internal dimension, both scenarios are equivalent.

MLDS requires observers to compare interval differences, i.e., which of two pairs of stimuli include the bigger difference (triads or quadruples). Figure A4a shows an example for a triad comparison, where the observer judges whether the interval Ψ(*s*_9_) − Ψ(*s*_4_) or Ψ(*s*_4_) − Ψ(*s*_2_) is perceived as bigger. Judgments are repeated several times to obtain a relative frequency of choosing one interval or the other. This frequency indicates the perceived magnitude of the intervals. A frequency of 0.5 indicates that both intervals are perceived to be approximately equal. A frequency close to 1 would indicate that the second interval is perceived as larger than the first. In the example in Figure A4a, the relative frequency is approximately 0.65, so the second interval is judged to be slightly greater than the first. By repeatedly presenting different triads and putting them all in the same statistical model, MLDS estimates the parameters for Ψ(*s*) that best explain the data (maximize the likelihood of the data).

A key aspect of MLDS is that observers judge perceptual *intervals*. These judgments contain easy and difficult comparisons, and hence produce relative frequencies across the entire range from ceiling (*p* = 0 or *p* = 1) to guessing (*p* = 0.5).

Pairwise comparisons of supra-threshold stimuli are easier than interval comparisons, and hence are more likely to result in ceiling frequencies. This would pose a problem for scale estimation because with frequencies close to one or zero are not informative about perceptual magnitudes and thus insufficient to constrain interval scales (Gescheider, 1997).

### How MLCM estimates perceptual scales from paired comparisons

To illustrate how MLCM estimates scales we use three selected pairwise comparisons (Figure A4b). The left panel shows two paired comparisons each within the same context and hence on the same perceptual scale. The first comparison is between *s*_4_ and *s*_5_ (black context). The stimuli elicit perceptual responses Ψ_*B*_(*s*_5_) and Ψ_*B*_(*s*_4_). The comparison is repeated several times and produces a relative frequency of judging *s*_5_ lighter than *s*_4_ of *p* = 0.75. The second comparison is between *s*_2_ and *s*_3_ (white context). *s*_3_ is perceived lighter than *s*_2_ with a relative frequency of *p* = 0.9. These two examples illustrate how the response frequencies determine the shape of each scale: the local slope of Ψ_*B*_ needs to be shallower than that of Ψ_*W*_ to produce a smaller perceptual interval between two stimuli (less discriminable). MLCM also includes comparisons across contexts. An example is shown in the right panel of Figure A4b. When *s*_4_ is shown in black and *s*_7_ in white, observers judge *s*_7_ as lighter with a relative frequency of *p* = 0.75. Because this frequency is the same as for the “within pair” (*s*_4_ and *s*_5_) in the black context, the perceptual interval should be of the same size. Considering all within- and across-context comparisons in the same statistical model, MLCM estimates scale values for which these interval relationships are preserved.

MLDS and MLCM can estimate scales only for relative frequencies that are not all zero or one. This was corroborated by our simulations with very low (unrealistic) noise levels. When either noise is too low or stimulus levels are too far apart from each other, the comparisons are not informative and the resulting MLCM scales show biases.
